# Iatrogenic bladder injury following gynecologic and obstetric surgery: A systematic review and meta‐analysis

**DOI:** 10.1111/aogs.14641

**Published:** 2023-08-08

**Authors:** Ann‐Sophie Jensen, Ina Isabell Kathleen Heinemeier, Jeppe Bennekou Schroll, Martin Rudnicki

**Affiliations:** ^1^ Department of Obstetrics and Gynecology Odense University Hospital Odense Denmark; ^2^ Department of Clinical Research, Faculty of Health Science University of Southern Denmark Odense Denmark; ^3^ Center for Evidence‐Based Medicine Odense (CEBMO) and Cochrane Denmark University of Southern Denmark Odense Denmark; ^4^ Department of Clinical Research University of Southern Denmark Odense Denmark; ^5^ Open Patient Data Exploratory Network, OPEN Odense University Hospital Odense Denmark

**Keywords:** bladder injury, iatrogenic, obstetric, surgery, gynecologic

## Abstract

**Introduction:**

Iatrogenic bladder injury is a rare complication following obstetric and gynecologic surgery and only sparse information is available regarding length of transurethral catheterization following injuries, suturing techniques including choice of suture, and complications. The primary aim of this systematic review was to evaluate length of transurethral catheterization in relation to complications following iatrogenic bladder injury. Second, we aimed to evaluate the number of complications following repair of iatrogenic bladder injuries and to describe suture technique and best choice of suture.

**Material and methods:**

A systematic review and meta‐analysis was conducted, and the results were presented in accordance with the Preferred Reporting Items for Systematic Reviews and Meta‐Analyses (PRISMA) guidelines. PubMed, Embase, and Medline electronic databases were searched, and followed by screening from two independent reviewers. Studies published between January 2000 and March 2023 describing methods of bladder injury repair following obstetric or gynecologic benign surgery were included. Data extraction was done using Covidence. We performed a meta‐analysis on complications after repair and explored this with a meta‐regression analysis (Metafor package R) on length of catheterization to determine if length of catheterization influenced the risk of complication. A risk of bias tool from Cochrane was used to assess risk of bias and the study was registered in PROSPERO (CRD42021290586).

**Results:**

Out of 2175 articles, we included 21 retrospective studies, four prospective studies, and one case‐control study. In total, 595 bladder injuries were included. Cesarean section was the most prominent surgery type, followed by laparoscopically assisted vaginal hysterectomy. We found no statistically significant association between length of transurethral catheterization and numbers of complications following repair of iatrogenic bladder injuries. More than 90% of injuries were recognized intraoperatively. Approximately 1% had complications following iatrogenic bladder injury repair (0.010, 95% confidence interval 0.0015–0.0189, 26 studies, 595 participants, *I* ^2^ = 4%).

**Conclusions:**

Our review did not identify conclusive evidence on the length of postoperative catheterization following bladder injury warranting further research. However, the rate of complications was low following iatrogenic bladder injury with a wide range of repair approaches.

## Graphical abstract

Our review did not reveal any beneficial effect of prolonged catheterization following surgery for iatrogenic bladder injury (*p* = 0.2341). The optimal catheterization length may be associated with the place and size of the injury and so far the optimal length of catheterization has not been clarified, even though it has a severe impact on life quality.

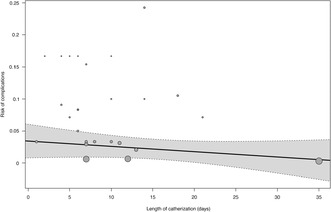

**Key message**

The optimal length of catheterization following iatrogenic bladder injury remains to be identified. However, the number of complications is low and need for reoperation is limited.

## 1 INTRODUCTION

Iatrogenic bladder injury is a known complication for gynecologic and obstetric surgery due to the anatomical relationship between the urinary tract organs and the female internal genitalia.^1^ The incidence of bladder injuries varies among procedures and surgical approaches. In a systematic review from 2018, Wong et al. reported an incidence of 0.24% of bladder injuries in gynecologic laparoscopy for benign indication.^1^ Other studies have reported an incidence of 0.44% during cesarean section^2^ and an incidence of 1.54% during hysterectomy for benign disorders.^3^ Multiple factors, such as previous pelvic or abdominal surgery, adhesions, endometriosis, and urinary tract abnormalities are associated with an increased risk of iatrogenic bladder injury during surgery.^1^, ^2^, ^4^

Lower urinary tract injuries, including bladder injuries, are associated with an increased morbidity. This includes fistula formation, infections, and renal failure. Furthermore, there is a high risk of reoperation (60%) following injuries to the urogenital system, and these injuries affect the length of hospitalization and quality of life.^1^, ^5^

Previous studies have reported varied types of sutures in the management of an iatrogenic bladder injury. If diagnosed intraoperatively, bladder injuries are most often repaired by a two‐layer suture followed by transurethral catheter drainage.^2^, ^6^ However, no consensus has been reached regarding suture type and suture size in the available literature.

Little is reported about the length of catheterization following iatrogenic urinary tract injury, but catheterization time ranges from 5 to 14 days in previous reports.^5^, ^6^ It is known that use of catheters increases the risk of urinary tract infections.^1^ It is critical to establish knowledge about the length of transurethral catheterization required to avoid reoperation and fistula formation, but also to eliminate unnecessary risk of urinary tract infections, and impact on patient's quality of life.

The aim of this systematic review was to conduct a comprehensive systematic review to evaluate the length of transurethral catheterization following iatrogenic urinary bladder injury in case of benign surgery. Second, we aimed to evaluate the number of complications following repair of iatrogenic bladder injuries, and to describe suture technique and best choice of suture.

## 2 MATERIAL AND METHODS

### 2.1 Data sources

Studies were identified by searching PubMed and Embase electronic databases during the period January 1, 2000 through to March 2023 (search date March 5, 2023). We used a combination of medical subject heading (MeSH) and single‐term search strategies. The following keywords and MeSH search‐terms were used: “Urinary tract injury”, “bladder injury/rupture/laceration/trauma”, “gynecology”, “obstetrics”, and “surgery”. Furthermore, we manually searched and reviewed references and bibliographies from relevant articles to identify additional sources. Our search was assisted by a search specialist at the University of Southern Denmark.

Duplicates were removed using EndNote's duplication filters. Then, literature was screened by two independent reviewers (A‐SJ, IIKH) to identify studies for inclusion. Data extraction was done independently and individually and then compared between the two reviewers. Data extraction was done using Covidence, developed for systematic reviews by the Cochrane Collaboration.^7^ In case of any discrepancies between extracted data, a third reviewer (MR) was consulted until consensus was obtained.

### 2.2 Main outcome measures

The primary outcome was the length of transurethral catheterization in days. Secondary measures were the number of complications following iatrogenic bladder injury repair. The complications included number of fistulas developed, renal complications, infections, and the reoperation rate. Furthermore, secondary measures were management of repair including suture type and suture size, number of suture layers, and interrupted or continuous sutures.

### 2.3 Eligibility criteria

Randomized controlled trials and observational studies on women with a bladder injury published between January 2000 and March 2023 were considered for inclusion. We included articles that described methods of bladder injury repair following obstetric or gynecologic benign surgery. Only original research was considered for inclusion. Excluded were bladder injuries after surgery for malignant indication, non‐human research, and studies including men and pediatric patients. In addition, reviews, conference abstracts, case reports, duplicate articles, unpublished articles, and articles written in languages other than English were excluded.

Risk of bias of the studies including assessment of bias for the observational studies was performed using GRADE.^8^ The systematic review is in accordance with the Preferred Reporting Items for Systematic Reviews and Meta‐Analyses (PRISMA) reporting guideline.^9^

### 2.4 Data collection and analysis

Demographics on the studies were collected and included: title, author, year of publication, country, study design, number of bladder injuries, and type of surgery. Numerical data including length of transurethral catheterization in days, number of complications including fistula formation, urinary tract infections, renal complications, and reoperation rate were collected. In addition, categorical data on suture type, suture size, number of suture layers, and intraoperative or postoperative recognition were collected.

A meta‐analysis was planned using the ‘metafor’ package in R to produce a forest plot to estimate the proportion of complications. We planned to examine whether the proportion of complications could be affected by number of average days of catheterization. This was done through a meta‐regression where average days of catheterization was used as covariate and the association was plotted on a graph. Examination of the association was made through the standard statistical test offered by the ‘metafor’ package. Heterogeneity was estimated by *I* ^2^ statistic. High heterogeneity was indicated if the *I* ^2^ value was above 75%.

The systematic review was registered in Prospero on November 24, 2021 (Reference ID: CRD42021290586).

## 3 RESULTS

**FIGURE 1:**
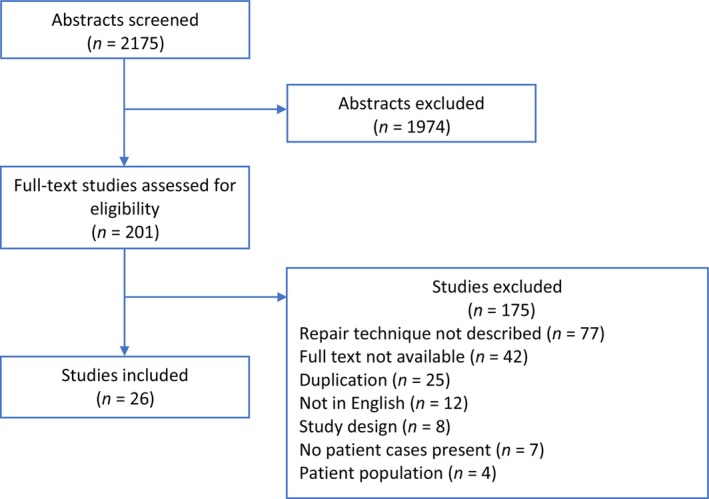
Systematic review selection process.

Our search revealed 2175 titles and abstracts for evaluation. Manual search including references from previous studies provided two additional studies. From title and abstract screening, 1974 studies were found to be irrelevant. In total, 201 studies were assessed for eligibility. Of these, 175 studies were excluded, leaving 26 studies for inclusion.^2^, ^4^, ^6^, ^10^, ^11^, ^12^, ^13^, ^14^, ^15^, ^16^, ^17^, ^18^, ^19^, ^20^, ^21^, ^22^, ^23^, ^24^, ^25^, ^26^, ^27^, ^28^, ^29^, ^30^, ^31^, ^32^ Exclusion was due to the following reasons: bladder repair technique not described (*n* = 77), full text not available (*n* = 42), duplication (*n* = 25), language (*n* = 12), study design (*n* = 8), no bladder injuries present (*n* = 7), and wrong patient population (*n* = 4) (Figure 1).

The included studies comprised 21 retrospective studies, four prospective studies, and one case‐control study (Table 1). In total, the studies included 595 iatrogenic bladder injuries following gynecologic or obstetric surgery. Of these, 48 injuries occurred during incontinence surgery (8.0%), 74 injuries during laparoscopic surgery (12.4%), and 439 injuries during open surgery (73.8%). The remaining 34 injuries were not classified (5.7%) (Table 1).

**TABLE 1:** Characteristics of included studies.

No	Study	Country	Design	Surgery type	Number of bladder injuries	Length of transurethral catheterization (days)	Number of complications	Complication
		Year						
1	Abdel‐Fattah^10^	UK 2006	Retrospective	Incontinence	2	10	N/A	
2	Abouassaly^11^	Canada 2004	Retrospective	Incontinence	14	1–7	0	
3	Anpalagan^12^	Australia 2011	Retrospective	Laparoscopic	14	4–10	0	
4	Aydin^4^	Turkey 2020	Retrospective	Laparoscopic	9	5–7	0	
5	Baggish^13^	USA 2010	Retrospective	Laparoscopic	33	7–21	8	3 Vesico vaginal fistulas 5 Urinomas
6	Buca^14^	Italy 2018	Retrospective	Incontinence	4	10	0	
7	Chamsy^15^	USA 2015	Retrospective	Laparoscopic	14	10	0	
8	Dolanbay^16^	Turkey 2016	Retrospective	Laparoscopic	2	5	0	
9	Franchia^17^	Italy 2019	Retrospective	Open	23	10–15	0	
10	Gellhaus^18^	USA 2014	Retrospective	N/A	2	4.1	N/A	
11	Gilani^19^	Pakistan 2020	Retrospective	Open	6	21	0	
12	Gungorduk^20^	Turkey 2010	Case‐control	Open	76	10–14	0	
13	Hammad^21^	United Arabic Emirates 2010	Retrospective	Open	32	9–14	1	1 Vesico vaginal fistula
14	Kato^22^	Japan 2009	Prospective	Incontinence	11	3–5	0	
15	Mahmood^23^	Pakistan 2017	Prospective	Open	161	28–42	0	
16	Sankareswari^24^	India 2017	Retrospective	Incontinence Laparoscopic Open	19	14–21	2	2 Vesico vaginal fistulas
17	Popovic^25^	France 2007	Retrospective	Incontinence	5	3–8	0	
18	Rahman^2^	Saudi Arabia 2008	Retrospective	Open	34	7	1	1 Unsuccessful repair
19	Sabadell^26^	Spain 2011	Retrospective	Incontinence	2	2	N/A	
20	Safrai^27^	Israel 2020	Retrospective	Open	14	7.7	0	
21	Salman^28^	Israel 2017	Retrospective	Open	81	5–10	0	
22	Sendag^29^	Turkey 2013	Retrospective	Laparoscopic	2	5–7	0	
23	Simsek^30^	Turkey 2014	Prospective	Incontinence	4	14	0	
24	Skorupska^31^	Poland 2020	Prospective	Incontinence	6	5	N/A	
25	Tian^32^	Taiwan 2007	Retrospective	Incontinence Laparoscopic	13	7	1	1 Vesico vaginal fistula
26	Yossepowitch^6^	Israel 2004	Retrospective	Open	12	4–9	1	1 Vecico vaginal fistula
Total					630		15	

Abbreviation: N/A, non applicable.

Among the injuries, 542 were recognized intraoperatively (91.1%), 37 postoperatively (6.2%), and 16 were not categorized (2.7%). The localization of injury was described in 295 women: bladder dome (*n* = 191), posterior wall (*n* = 54), anterior wall (*n* = 37), both anterior and posterior walls (*n* = 4), trigone (*n* = 4), and paravesical fossa (*n* = 5). The localization of injury was not specified in the remaining women.

**FIGURE 2:**
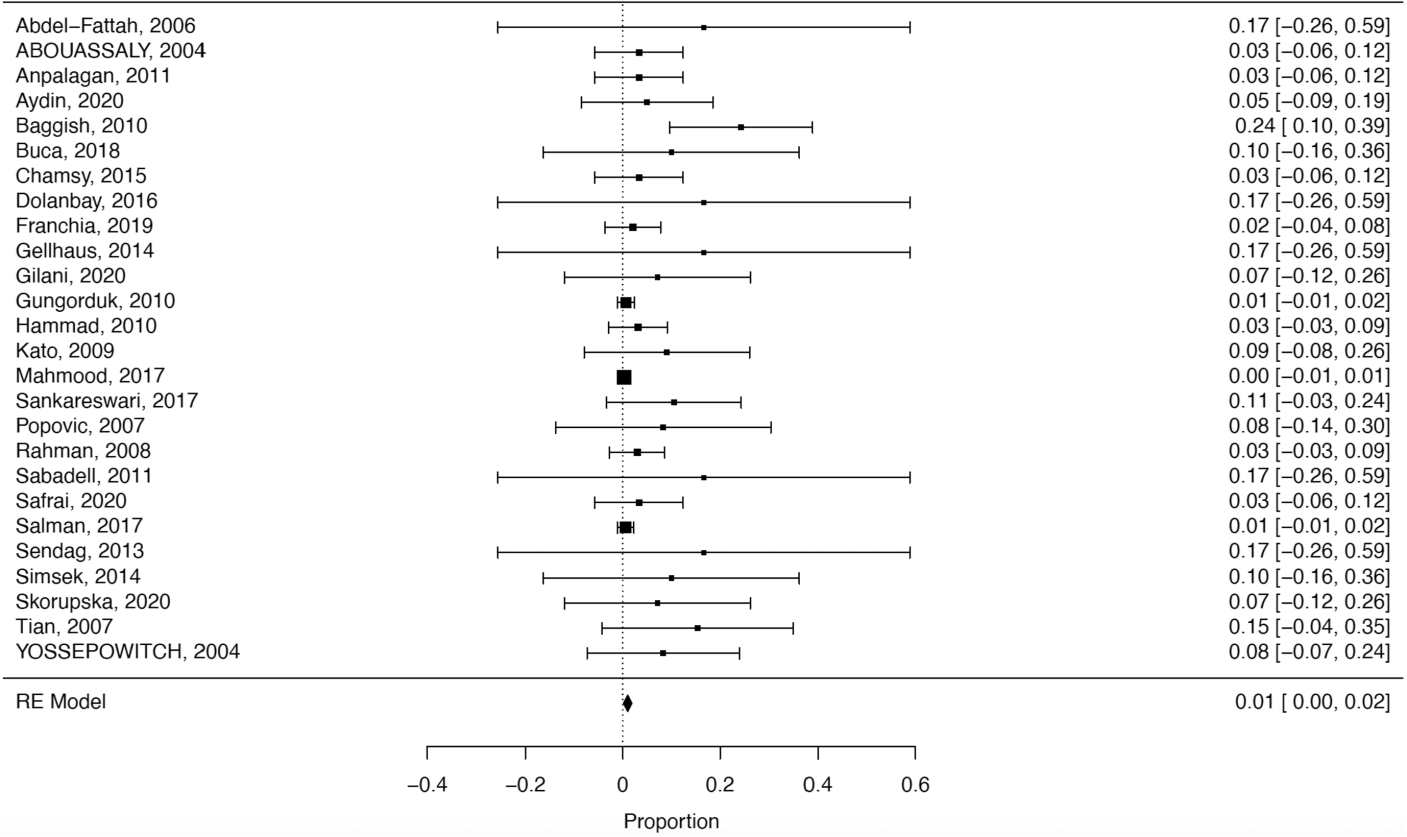
Forest plot of the included studies showing the proportion of complications following iatrogenic bladder injury (0.010, 95% confidence interval 0.0015–0.0189, 26 studies, 595 participants, *I* ^2^ = 4%).

**FIGURE 3:**
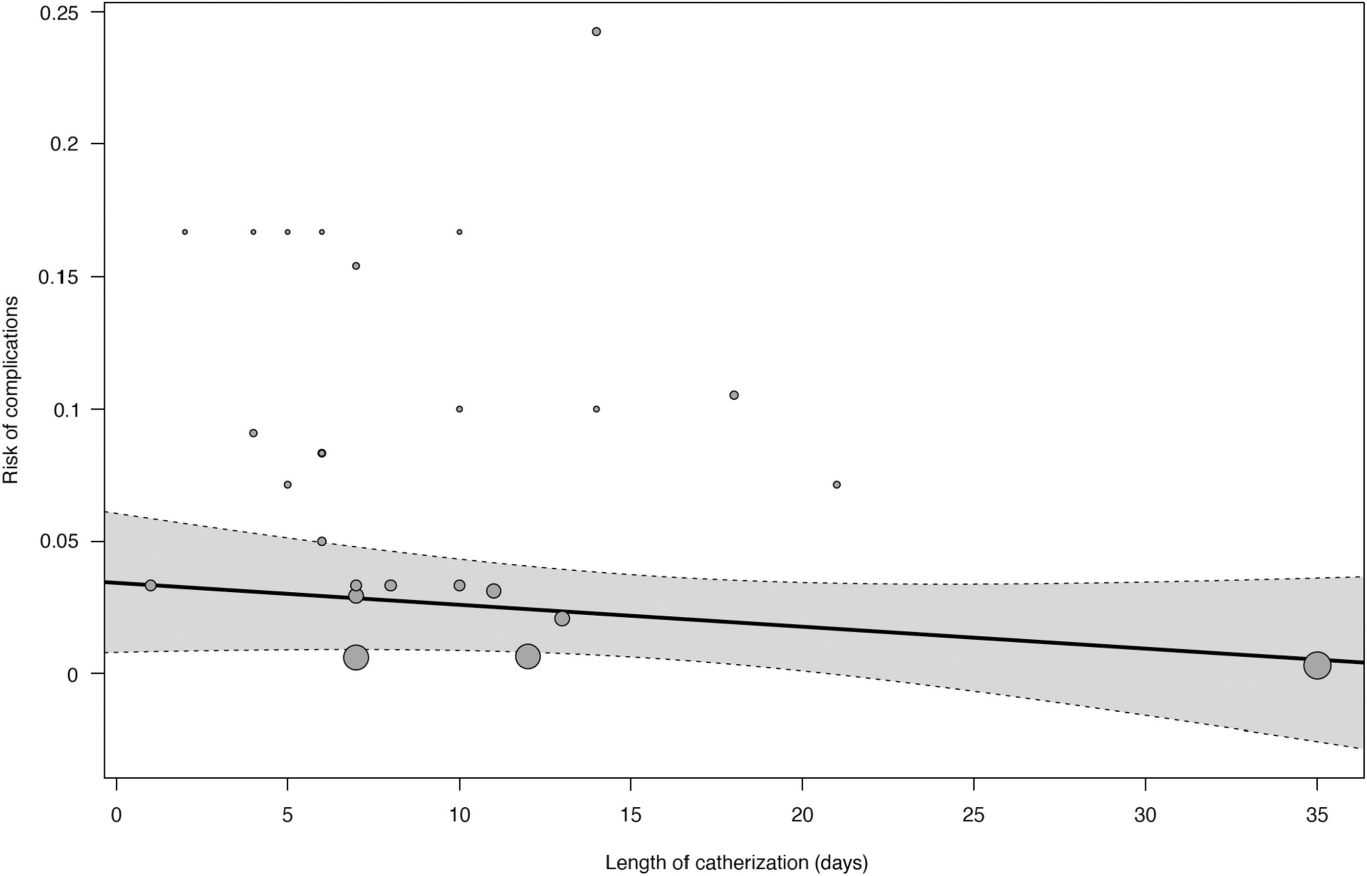
Meta‐regression analysis showing complications as a function of average length of urinary catheterization (*p* = 0.2341).

The length of transurethral catheterization was described in 26 studies and ranged from 1 to 42 days. Figure 2 shows that the proportion of bladder injuries in the included studies was 0.010 (95% confidence interval [CI] 0.0015–0.0189, 26 studies, 595 participants, *I* ^2^ = 4%). To investigate a possible correlation between length of catheterization and number of complications we conducted a meta‐regression analysis. The meta‐regression analysis showed no statistically significant correlation between the length of transurethral catheterization and the number of complications (*p* = 0.23, Figures 2 and 3).

In total, 14 complications were registered (2.4%). Vesicovaginal fistula was the most frequently observed complication (eight women, 57.1%), urinomas in five women (35.7%), and unsuccessful repair in one woman (7.1%).^2^ No urinary tract injuries were registered. One fistula developed following cesarean section,^6^ three following laparoscopic surgery,^13^ and in the remaining four, the primary surgical method was not described.^21^, ^24^, ^32^ In addition, the surgical method was not described in the five women with urinomas.^13^

In total, seven of the fistulas (87%) required reoperation. In the remaining case, it was not reported if reoperation was needed. Our meta‐analysis disclosed the risk of complications at approximately 1% (0.010, 95% CI 0.0015–0.0189, 26 studies, 595 participants, *I* ^2^ = 4%).

Table 2 shows the characteristics of management of bladder injury repair. In total, 14 studies described the suture type (53.8%). In four studies, the injuries were managed conservatively without suturing with permanent catheter for 1–14 days (14.3%). Eight studies did not describe the suture type (30.8%). The suture material was listed in four studies as Vicryl (15.4%), in two studies as Chromic catgut (7.7%), as Polyglycolic acid in one study (3.8%), and absorbable or delayed absorbable sutures in three studies (11.5%). The remaining four studies used different suture materials. One study used Vicryl in nine women and Dexon in one woman.^12^ Another study used Chromic Catgut when the injury was repaired by a urologist, Polyglycolic acid when repaired by a gynecologist, and two bladder injuries were repaired with permanent suture.^13^ Vicryl or Polyglactin was used in another study.^4^ Finally, one study used different sutures depending on the injury. Full‐thickness injuries were repaired with barbed suture or smooth delayed absorbable suture.^17^

**TABLE 2:** Management of bladder injury repair

Study	Number of bladder injuries	Suture size	Suture type	Intraoperative detection, *n*
Abdel‐Fattah^10^	2	N/A	N/A	1
Abouassaly^11^	14	Not sutured	Not sutured	14
Anpalagan^12^	14	2.0/3.0	Polyglactin (incl. Vicryl) Polyglycolic acid suture (incl. Dexon)	14
Aydin^4^	9	3.0/4.0	Polyglactin (incl. Vicryl)	9
Baggish^13^	33	2.0	Chromic catgut Polyglycolic acid suture (incl. Dexon) Permanent suture	19
Buca^14^	4	Not sutured	Not sutured	4
Chamsy^15^	14	2.0	Barbed suture Smooth delayed absorbable suture	
Dolanbay^16^	2	N/A	Absorbable	
Franchia^17^	23	3.0/4.0	Absorbable Delayed absorbable	23
Gellhaus^18^	2	2.0/3.0	Polyglactin (incl. Vicryl)	2
Gilani^19^	6	2.0	Polyglactin (incl. Vicryl)	6
Gungorduk^20^	76	N/A	N/A	76
Hammad^21^	32	N/A	N/A	32
Kato^22^	11	N/A	N/A	11
Mahmood^23^	161	2.0	Polyglactin (incl. Vicryl)	145
Sankareswari^24^	19	N/A	N/A	17
Popovic^25^	5	N/A	N/A	5
Rahman^2^	34	2.0	Chromic catgut	34
Sabadell^26^	2	Not sutured	Not sutured	2
Safrai^27^	14	N/A	N/A	14
Salman^28^	81	2.0	Polyglactin (incl. Vicryl)	78
Sendag^29^	2	3.0	Polyglycolic acid suture (incl. Dexon)	2
Simsek^30^	4	Not sutured	Not sutured	4
Skorupsa^31^	6	N/A	N/A	6
Tian^32^	13	3.0	Chromic catgut	12
Yossepowitch^6^	12	N/A	Absorbable	12

Abbreviation: N/A, non applicable.

The suture size was described in 12 studies. Six studies used 2.0 sutures (23.1%), two studies used 3.0 sutures (7.7%), two studies used either 2.0 or 3.0 sutures (7.7%), and two studies used either 3.0 or 4.0 sutures (7.7%) (Table 2). Of these, one study used interrupted sutures,^4^ and one study used continuous sutures.^6^ Use of both interrupted and continuous sutures was reported by one study.^15^ Another study reported use of continuous sutures when repaired by urologist but did not specify suture type when repair was by a gynecologist.^13^ Finally, one study reported that the mucosal layer was closed with continuous suture, whereas the submucosal and muscular layers were closed with continuous or interrupted sutures with the inversion of the mucosal layer.^17^

The studies included a high risk of bias following the GRADE approach. All studies were observational studies referred to as low‐quality evidence. Some studies were downgraded to very low quality because of missing internal controls and failure of measuring known important prognostic factors.

## 4 DISCUSSION

In our systematic review we found that the length of transurethral catheterization ranged from 1 to 42 days. In total, the included studies reported 14 complications, where vesicovaginal fistula formation was the most frequently reported. We found no correlation between the length of transurethral catheterization and the number of complications.

Bladder healing is a multistep process involving hemostasis, inflammation, cellular proliferation, extracellular matrix, and remodeling including other factors such as MicroRNAs.^33^ Normally, the damaged tissue is replaced through proliferation and cellular migration following the hemostasis and inflammation steps. Normal tissue matures with scar formation when the proliferation stops.^33^ The human urinary bladder has the capacity to restore both anatomy and functionality even after subtotal cystectomy. A previous study in rodents showed that the urothelium proliferated actively between the second and third days. Furthermore, they found that the inflammatory response is related to the injury, and all animals in the study had no voiding difficulties subsequently. However, the healing process may be delayed in complicated cases such as fistula where the defect has been present for a longer period.^34^ A previous report suggested that transurethral catheterization may last 2–3 weeks to ensure that the bladder has healed sufficiently.^39^ On the other hand, a study from Norway showed sufficient healing after 14 days of catheterization after fistulation due to obstetric fistula after delivering^35^ and similarly a recent randomized controlled trial disclosed that 7‐day bladder catheterization after repair of simple fistula is non‐inferior to 14‐day catheterization, suggesting that even in complicated cases catheterization for more than 7 days may not be needed.^36^ However, in the studies included in this review the catheterization length ranged from 1 to 42 days, and it was not specified how long catheterization lasted in the women with fistulas following primary repair. Our meta‐regression analysis showed no statistically significant correlation between the length of transurethral catheterization and the number of complications.

Our comprehensive review of the literature from the past 20 years shows that the most frequently used suture type, when repairing iatrogenic bladder injury, was Vicryl® followed by Chromic Catgut®. Most countries no longer use Chromic Catgut because of its origin from bovines, and the presence of this suture probably reflects that our literature search included the last 20 years. However, looking at the current practice at our department it appears that barbed sutures have been introduced without any documentation and irrespective of the size of lesion. The use of barbed sutures may be due to the ease of suturing by laparoscopy.

Cesarean section was the procedure that accounted for the highest number of bladder injuries. In our review, 250 (39.7%) of the bladder injuries occurred during cesarean sections. A systematic review recently identified that the number of cesarean sections has increased in recent years as a result of scientific progress, and social and cultural changes.^37^ Therefore, it is crucial for obstetricians to keep in mind that bladder injuries are potential complications of cesarean sections. Additionally, it is important that the surgeon knows how to manage an iatrogenic bladder injury and possesses adequate knowledge of bladder healing processes. Furthermore, it is of importance to offer a safe but not too long catheterization period following bladder injuries due to cesarean section. Whether this may be as short as 7 days or longer remains to be clarified and probably the location of the injury may also be taken into consideration.

It has been stated that sufficient knowledge of abdominal and pelvic anatomy can prevent iatrogenic bladder injuries. Therefore, the surgeons' preferences and experiences are of great importance when keeping the incidence of iatrogenic bladder injuries at its lowest due to inconsistency in the literature on management of bladder injuries. In addition, this supports the importance of this systematic review.

Most of the included studies were retrospective designs (80.1%). It is likely that an underreporting of bladder injuries occurs within retrospective research, highlighting the need for prospective research. A previous study found that intraoperative cystoscopy increased the intraoperative detection rate of bladder injury, suggesting that use of cystoscopy may be a tool to further increase the intraoperative detection rate.^38^ However, the intraoperative detection rate was 91.1% in this systematic review, despite the use of intraoperative cystoscopy only being reported in 10 (38.5%) of the included studies. The limited use of cystoscopy may reduce the overall intraoperative detection rate in this systematic review, although the demonstrated detection rate was high.

Our systematic review was conducted from all available published studies of iatrogenic bladder injuries following gynecologic and obstetric surgery for benign indications within the last 23 years. The number of studies included, therefore, led to a large number of women. Incomplete reporting of the management of an iatrogenic bladder injury, however, may limit our understanding of bladder injury repair.

A limitation of this review is that the studies included are low to very low quality and the fact that the studies differ by surgical approach, leaving a high clinical heterogeneity. However, we only included benign cases and excluded malignant cases, which may contribute to reduce the heterogeneity. Furthermore, we cannot exclude that more severe lesions have been associated with longer periods of catheterization whereas small lesions have been treated with shorter time of catheterization, explaining that few complications were observed. However, this needs to be clarified. Our review demonstrates the need for further research regarding repair of iatrogenic bladder injuries to understand repair by surgical approach including length of catheterization, suture type, and suture size.

## 5 CONCLUSION

Our review did not disclose any specific time of catheterization following bladder injury and the length of catheterization had no impact on complications following bladder injuries. From a practical point of view, the size and place of injury may be of relevance for the length of catheterization, but this depends on future studies. Further, our review represents a comprehensive summary of the management of iatrogenic bladder injury in obstetric and gynecologic surgery for benign indication.

## AUTHOR CONTRIBUTIONS

ASJ, IIK Hinemeier, MR: Design and data collection. ASJ, IIKH, JBS, MR: Data evaluation and writing the manuscript.

## FUNDING INFORMATION

None.

## CONFLICT OF INTEREST STATEMENT

The authors have stated explicitly that there are no conflicts of interest.
